# Current state of heart failure treatment: are mesenchymal stem cells and their exosomes a future therapy?

**DOI:** 10.3389/fcvm.2025.1518036

**Published:** 2025-04-28

**Authors:** Chengqian Chen, Wentao Zhong, Hao Zheng, Wei Zhao, Yushi Wang, Botao Shen

**Affiliations:** ^1^ Department of Cardiology Center, The First Hospital of Jilin University, Changchun, China; ^2^ Department of Endocrinology and Metabolism, The First Hospital of Jilin University, Changchun, China

**Keywords:** heart failure, treatment, mesenchymal stem cells, exosomes, cell transplantation

## Abstract

Heart failure (HF) represents the terminal stage of cardiovascular disease and remains a leading cause of mortality. Epidemiological studies indicate a high prevalence and mortality rate of HF globally. Current treatment options primarily include pharmacological and non-pharmacological approaches. With the development of mesenchymal stem cell (MSC) transplantation technology, increasing research has shown that stem cell therapy and exosomes derived from these cells hold promise for repairing damaged myocardium and improving cardiac function, becoming a hot topic in clinical treatment for HF. However, this approach also presents certain limitations. This review summarizes the mechanisms of HF, current treatment strategies, and the latest progress in the application of MSCs and their exosomes in HF therapy.

## 1 Introduction

Heart failure (HF) is a clinical syndrome caused by impaired systolic and/or diastolic function of the heart, leading to pulmonary and/or systemic congestion and inadequate tissue perfusion. It primarily manifests as dyspnea, limited physical activity, and fluid retention (1, 2). The pathophysiology of HF is complex and involves multiple neurohormonal pathways, including the renin-angiotensin-aldosterone system (RAAS), sympathetic nervous system, and natriuretic peptide system (1, 3–5). The RAAS plays a central role in the pathogenesis and progression of HF, where activation of circulating and/or tissue RAAS leads to sodium and water retention, vasoconstriction, myocardial and vascular hypertrophy, fibrosis, and remodeling while enhancing the detrimental effects of other neurohormonal systems, such as the sympathetic nervous system (6–9). Heart failure is a terminal manifestation of cardiovascular disease and remains the primary cause of death, posing one of the two major challenges in cardiovascular medicine in the 21st century. According to epidemiological surveys, HF is rapidly emerging as a global public health issue, affecting over 64 million people worldwide. The incidence and mortality of HF continue to rise each year, placing a significant burden on healthcare systems (10). In Western countries, 1%–2% of hospitalizations are due to HF, making it the most common cause of hospital admission for patients over 65 years old. Approximately 30%–40% of HF patients have a history of hospitalization, with 50% being readmitted within a year of the initial diagnosis. HF is associated with high mortality, with 1-year mortality rates ranging from 15%–30% in certain populations and 5-year mortality rates reaching up to 75% (10). In Western countries, the annual healthcare cost per HF patient can reach €25,000, and the rising prevalence of HF is expected to further escalate these costs, even for developed nations (10). The expanding population of HF patients and the associated complications severely impact patients' quality of life. Current clinical treatments for HF primarily include pharmacological and non-pharmacological approaches. Traditional pharmacotherapy involves diuretics, β-blockers, angiotensin-converting enzyme inhibitors (ACEI) or angiotensin receptor blockers (ARB), and inotropic agents (11). Despite recent advancements in pharmacotherapy, traditional treatments still face certain limitations (12, 13). Non-pharmacological treatments mainly include interventions like cardiac resynchronization therapy (CRT) and implantable cardioverter-defibrillators (ICD), which have improved survival rates in some patients, though long-term prognosis remains poor (14, 15). Meanwhile, heart transplantation has offered hope for some critically ill patients, but challenges remain, including limited donor availability, severe post-operative complications, and high medical costs (16–18). Given that myocardial cells are permanent and non-regenerative, damaged myocardial cells and their microenvironment progressively deteriorate (19). Traditional pharmacological and non-pharmacological treatments can only alleviate symptoms but cannot reverse myocardial degeneration and necrosis or improve cardiac function fundamentally. With advancements in regenerative medicine, stem cell transplantation has emerged as a promising field. Under optimal conditions, stem cells can either differentiate directly into functional cardiomyocytes or indirectly promote cell survival through the secretion of exosomes. As such, stem cell-derived cardiomyocytes may replace dead myocardial cells and restore cardiac function, offering a novel and promising approach to HF treatment. Numerous studies have demonstrated the feasibility of MSCs transplantation for HF treatment. This review summarizes the mechanisms of HF, the current treatment landscape, and the latest developments in MSCs and exosome therapies for HF.

## 2 Mechanisms of heart failure

The most fundamental contraction of the heart is regulated by calcium ions. Calcium is released from the sarcoplasmic reticulum through ryanodine receptors, and the elevated intracellular Ca2+ concentration activates cardiac contraction. During diastole, Ca2+ is transported back into the sarcoplasmic reticulum via Ca2+-ATPase and sequestered. In HF, the activity of Ca2+-ATPase is reduced, leading to decreased uptake and storage of Ca2+ in the sarcoplasmic reticulum, which impairs cardiac repolarization and weakens myocardial contractility (20). Some studies indicate that increasing Ca2+ levels can enhance contractility in HF patients to some extent, yet other studies have shown that elevated Ca2+ can damage elastin in cardiomyocytes, increasing the risks of arrhythmia, myocardial hypertrophy, and apoptosis (21). These findings suggest that calcium dysregulation plays a key role in the pathogenesis of HF. Disrupted Ca2+ transport and homeostasis are associated with the development and progression of various diseases, including HF (22). Research also points out that serum Na+ and Ca2+ levels in HF patients are reduced, potentially contributing to impaired contractility and abnormal potassium channel expression, which exacerbates ventricular remodeling and leads to HF (23). Moreover, studies have revealed that the disruption of mitochondrial-sarcoplasmic reticulum junctions contributes to sinoatrial node dysfunction in HF, indicating that sarcoplasmic reticulum restructuring is involved in HF pathogenesis, further implying Ca2+ conversion abnormalities (24).

Oxidative stress (OS) has been demonstrated at every stage of HF development, and excess reactive oxygen species (ROS) can promote myocardial fibrosis, leading to cardiac remodeling, which in turn leads to HF and is involved in the progression of HF (25–27). ROS can be produced in the heart through various sources, including mitochondria, NADPH oxidase, uncoupled NO synthase (NOS), xanthine oxidase, cytochrome P450, and catecholamine autoxidation (28, 29). Excessive ROS production leads to mitochondrial oxidative stress and triggers mitochondrial dysfunction, including impairments in mitochondrial biogenesis, fatty acid metabolism, and antioxidant defense mechanisms. Increased mitochondrial ROS production has been demonstrated in myocardial cells in experimental models of HF and myocardial infarction (MI) (30). Furthermore, studies show that STAT3, a redox gene, protects mitochondrial integrity in cardiomyocytes, suppresses ROS formation, and regulates the expression of target genes like ErbB and Bcl-xL. However, STAT3 expression is reduced in damaged cardiomyocytes, which promotes cell death and fibrosis (31–33).

Inflammatory responses are crucial in the myocardial injury repair process. Leukocytes participate in the clearance of necrotic cardiomyocytes and initiate the myocardial repair process. Cardiomyocytes release adenosine triphosphate (ATP), which recruits macrophages to the site of inflammation to remove dead cells and debris (34). As macrophages digest apoptotic cells, various inflammatory cytokines, such as Tumor necrosis factor-α(TNF-α), Interleukin(IL)-1 β, IL-6, and transforming growth factor-β (TGF-β), may accumulate (35–37), promoting fibroblast activation, extracellular matrix (ECM) degradation, and increased matrix metalloproteinase (MMP) activity (38–40), further inducing cardiomyocyte apoptosis, autophagic damage, and fibrosis, eventually leading to cardiac hypertrophy (41). As ECM proteins are remodeled, the dead cardiomyocytes are replaced by scar tissue. Persistent inflammation, along with the continued infiltration of lymphocytes and macrophages into the scar tissue, exacerbates cardiac remodeling and reduces cardiac function, culminating in HF (41–43).

## 3 Current status of heart failure treatment

Traditionally, HF treatment includes general/basic therapy, pharmacological interventions, device-based therapies, and surgical interventions. In terms of general/basic therapy, patients must limit sodium intake (<5 g/day) and restrict fluid intake (<1–1.5 L/day) (44). Diuretics are used to alleviate symptoms of congestion, such as dyspnea and edema, with common medications including furosemide and spironolactone (45, 46). In recent years, the primary shift in HF pharmacotherapy has been towards the inhibition of excessive neurohormonal activation and its contribution to myocardial remodeling. Representative drugs include ACEI/ARB, β-blockers, and aldosterone receptor antagonists. In recent years, angiotensin receptor-neprilysin inhibitors (ARNIs) and sodium-glucose cotransporter-2 inhibitors (SGLT2i) have also emerged as prominent treatments. Renin-angiotensin system inhibitors, β-blockers, and aldosterone receptor antagonists can inhibit ventricular remodeling and reduce mortality in HF patients (47–50). ARNI, which combines ARB and enkephalinase inhibitor effects, raises levels of natriuretic peptides, bradykinin, and adrenomedullin, counteracting the vasoconstriction, sodium retention, and cardiac remodeling caused by excessive neurohormonal activation. The PARADIGM-HF trial demonstrated that sacubitril/valsartan sodium reduced the risk of the primary composite endpoint (cardiovascular death and HF hospitalization) by 20%, including a 20% reduction in sudden cardiac death, compared to enalapril (51). Subsequent studies such as TRANSITION and PIONEER-HF further validated the safety and efficacy of early initiation of ARNI therapy in hospitalized patients with acute decompensated HF (52, 53). SGLT2i, originally used as oral hypoglycemic agents by promoting the excretion of glucose and sodium through urine, have revealed unique cardiovascular effects. Studies have shown that SGLT2i reduces the risk of cardiovascular death and HF hospitalization in patients with type 2 diabetes (54), and their use has expanded to cardiovascular disease patients without diabetes. The DAPA-HF study confirmed that SGLT2i reduces the relative risk of HF worsening (hospitalization or emergency treatment with intravenous anti-HF medications) or cardiovascular death by 26% in HFrEF patients (55).

In addition to the aforementioned drugs, other medications like vericiguat, ivabradine, digitalis, and trimetazidine are also used in HF treatment. Vericiguat is the first oral soluble guanylate cyclase (sGC) stimulator approved for the treatment of symptomatic chronic HF with reduced ejection fraction (HFrEF) in adults (56). Vericiguat stimulates sGC independently of nitric oxide (NO) concentration and enhances NO sensitivity, increasing intracellular cyclic guanosine monophosphate (cGMP) levels, leading to smooth muscle relaxation and vasodilation, thereby improving cardiac structure and function. Increasing clinical studies have shown that vericiguat can reduce the incidence of cardiovascular death or HF hospitalization (57, 58). Ivabradine, by specifically inhibiting the cardiac pacemaker current in the sinoatrial node, reduces heart rate. The SHIFT study demonstrated that ivabradine could reduce the relative risk of cardiovascular death and hospitalization by 18%, while also significantly improving left ventricular function and quality of life in HF patients (59–61). Digitalis acts by inhibiting Na+/K+-ATPase, producing a positive inotropic effect, enhancing parasympathetic nerve activity, and reducing atrioventricular conduction. Studies have shown that long-term use of digoxin in HF patients does not affect mortality but reduces overall hospitalization rates and HF-related hospitalizations (62, 63). Trimetazidine improves myocardial mitochondrial function and glucose uptake through AMPK, alleviating pressure overload-induced HF (64). A recent meta-analysis demonstrated that trimetazidine significantly reduces cardiovascular mortality and HF hospitalization rates in HFrEF patients (65). Other emerging or experimental drugs include tafamidis for transthyretin amyloid cardiomyopathy (66), the myosin activator (omecamtiv mecarbil) (67) and the interleukin-1β inhibitor (canakinumab) (68), all of which hold promise for future clinical application.

Devices and surgical interventions also play a crucial role in HF treatment. Coronary artery bypass grafting (CABG) and percutaneous coronary intervention (PCI) can be performed to revascularize patients with HF. In the STICH trial, revascularization via CABG in patients with HFrEF was shown to reduce long-term mortality (69). However, in the REVIVED-BCIS2 trial, in patients with severe ischemic LV systolic dysfunction treated with optimal medications, revascularization by PCI did not reduce the incidence of death from any cause or hospitalization for HF (70). Currently, the most widely used cardiac implantable electronic devices mainly include CRT and ICD. CRT is used in HF patients with dyssynchronous ventricular contraction, improving atrioventricular and biventricular contraction synchrony, thus alleviating clinical symptoms, enhancing exercise tolerance and quality of life, reversing ventricular remodeling, improving cardiac function, and reducing HF-related hospitalization and mortality. It represents a milestone in device-based therapy for HF. Traditional biventricular pacing CRT has been used for the treatment of chronic HF for over 30 years. However, despite continual updates in CRT technology, approximately one-third of patients still experience treatment failure or non-response, resulting in no clinical benefit. In recent years, the emergence of conduction system pacing (CSP), as a new approach for CRT, has addressed some of these issues. CSP includes His bundle pacing (HBP) and left bundle branch area pacing (LBBAP), which directly stimulate the heart's native conduction system and offer greater benefits compared to traditional right ventricular pacing (71). Some recent clinical studies have demonstrated the feasibility and efficacy of CSP in CRT, showing not only improved synchrony of cardiac contraction in HF patients but also enhanced echocardiographic parameters and clinical outcomes (72–74). However, since CSP is a relatively new approach, its long-term clinical efficacy and safety still require further validation through large-scale, randomized controlled trials. The 2023 guidelines from the American Heart Rhythm Society raised the recommendation level for HBP and LBBAP in preventing and mitigating HF (75).CRT is limited to HF patients with widened QRS complexes and dyssynchronous ventricular contraction. The advent of the cardiac contractility modulation (CCM) device fills the gap in treating HF patients with narrow QRS complexes (76, 77). CCM is a novel device that treats chronic HF by delivering high-energy (approximately 7.5 V), long-duration (about 20 ms) electrical signals to the right ventricular septum during the absolute refractory period to enhance myocardial contractility. This stimulation does not generate an electrical activation in the myocardium but instead modulates calcium ion channels, increasing calcium ion regulation in cells, thus enhancing myocardial contractility and reversing ventricular remodeling (78). CCM represents a groundbreaking therapeutic device. Clinical studies have shown that CCM improves symptoms in HF patients, increases exercise tolerance, enhances quality of life, and reduces HF-related rehospitalization rates (79–82). However, there is a lack of prospective randomized controlled trials to assess whether long-term CCM therapy can reduce mortality in HF patients and improve long-term outcomes. It is also unclear whether the incidence of ventricular arrhythmias will increase, and further validation is needed. Moreover, all studies and trials on CCM have focused on left-sided HF, and no studies have yet evaluated the impact of CCM on patients with right-sided HF or diastolic HF, who currently lack effective treatment options. HF patients, particularly those with ischemic cardiomyopathy and significantly reduced left ventricular ejection fraction (LVEF ≤ 35%), face a high risk of sudden cardiac death (SCD) (83). Prophylactic ICD implantation can reduce the rate of SCD in HF patients (84). However, potential complications of traditional ICD implantation include device dislocation, pneumothorax, hematoma, and infection (85). To minimize these complications, the subcutaneous implantable cardioverter-defibrillator (S-ICD) has been developed. Compared to traditional ICDs, the S-ICD is safer, more effective, and more suitable for patients with difficulties in venous access, catheter-related complications, a high risk of infection, or those who are younger (86). Excessive sympathetic activation and reduced parasympathetic activity are compensatory mechanisms in HF that lead to cardiac damage, congestion, and decreased stroke volume. Therefore, neuromodulation has emerged as a new therapeutic approach for HF. Baroreflex activation therapy (BAT) involves electrical stimulation of the carotid baroreceptors, resulting in a centrally mediated reduction in sympathetic signals and an increase in parasympathetic signals, thereby improving the imbalance in autonomic regulation in HF patients and achieving therapeutic effects (87). Studies such as HOPE-4HF (88) and BeAT-HF (89) have confirmed that BAT significantly improves New York Heart Association (NYHA) functional classification, 6-min walk distance, and left ventricular ejection fraction (LVEF). Sleep apnea is a common comorbidity in HF patients, significantly affecting cardiovascular function (90). Sleep apnea can be classified into obstructive sleep apnea (OSA) and central sleep apnea (CSA).CSA is closely associated with HF, with 30%–50% of HF patients developing CSA, and its prevalence increases as HF severity worsens (91, 92). Phrenic nerve stimulation (PNS) has been shown to improve the apnea-hypopnea index and quality of life in CSA patients, while also demonstrating good safety (93, 94). Mechanical circulatory support (MCS) systems, particularly extracorporeal life support and extracorporeal membrane oxygenation (ECMO), can be used as “bridge therapies” for patients with acute and rapidly deteriorating HF or cardiogenic shock to stabilize hemodynamics, restore end-organ function, and buy time for further interventions such as heart transplantation or other MCS devices (95–97). For patients with end-stage HF, heart transplantation remains the only viable treatment option (16, 98).

Over the past few decades, significant advancements have been made in the treatment of HF. However, the prognosis for HF patients remains unsatisfactory. In the ESC-HF-LT study, the 1-year all-cause mortality rate for acute heart failure (AHF) was 23.6%, and for chronic heart failure, it was 6.4% (99). The combined endpoint of 1-year mortality or HF-related hospitalization was 36% for AHF and 14.5% for CHF (99). The 5-year survival rate for HF patients remains below 50% (100), and the mortality rate from HF is on the rise (101). There is still much to explore in the treatment of HF. Given that stem cells can differentiate into functional cardiomyocytes or enhance the survival of cardiomyocytes through paracrine functions, stem cell therapy holds potential for the treatment of HF.

## 4 Origin and biological characterization of mesenchymal stem cells

In the 1970s, Friedenstein et al. first described MSCs as spindle-shaped, adherent, non-hematopoietic stem cells located in mouse bone marrow (102). Later, in 1991, Caplan and colleagues named the cells isolated by Friedenstein as MSCs (103, 104). Since then, MSCs have become widely known as multipotent stem cells with self-renewal and immunomodulatory properties, attracting increasing attention from researchers due to their hidden clinical value. MSCs can be isolated from various adult tissues such as bone marrow, umbilical cord, placenta, adipose tissue, peripheral blood, liver, and dental pulp (105, 106), and they exhibit excellent self-renewal capacity *in vitro*, capable of persisting for over four months (107).

MSCs possess multipotent differentiation, low immunogenicity, immunomodulatory, and anti-inflammatory properties. When cultured MSCs are stimulated by certain cytokines, they can generate osteoblasts, chondrocytes, adipocytes, skeletal muscle cells, neurons, endothelial cells, and even cardiomyocytes (108–112). The International Society for Cellular Therapy (ISCT) has published the gold standard for MSCs: MSCs are distinguished from other cell types by expressing CD73, CD90, and CD105 on their surface while lacking expression of CD45, CD34, CD14, CD19, CD11b, and human leukocyte antigen (HLA)-DR (113, 114). Most importantly, MSCs exhibit low expression of major histocompatibility complex class II (MHC-II), the Fas ligand, and co-stimulatory moleculemolecules (B7-1, B7-2, CD40, and CD40l) on their surface (115, 116), indicating their low immunogenicity. Furthermore, MSCs inhibit the proliferation of T cells, B cells, natural killer (NK) cells, and dendritic cells (DCs). MSCs can block various immune cell functions, including cytokine secretion and cytotoxicity of T and NK cells, B cell maturation and antibody secretion, DC maturation and activation, and antigen presentation (117). This highlights the immunomodulatory activity of MSCs. MSCs protect tissues or organs from excessive inflammation through several mechanisms (118). First, MSCs express IL-1 receptor antagonist, downregulating IL-1 expression (119, 120). Second, MSCs establish a negative feedback loop in which macrophage-secreted TNF-α and other pro-inflammatory cytokines activate MSCs to secrete multifunctional anti-inflammatory proteins such as TNF-α-stimulated gene/protein 6 (TSG-6) and prostaglandin E2 (PGE2). TSG-6 reduces nuclear factor-*κ*B (NF-*κ*B) signaling in resident macrophages, and PGE2 stimulates the polarization of macrophages from a pro-inflammatory M1 phenotype to an anti-inflammatory M2 phenotype, thereby regulating the pro-inflammatory cytokine cascade (121). Third, MSCs establish a second negative feedback loop in which endotoxins, nitric oxide, or other damage-associated molecular patterns from injured tissues and macrophages activate MSCs to secrete PGE2, transforming macrophages into an IL-10-secreting phenotype (122). Thus, MSCs exhibit anti-inflammatory effects. In summary, the low immunogenicity, immunomodulatory, and anti-inflammatory properties of MSCs make them advantageous for cell therapy. These unique characteristics endow MSCs with potential and attractiveness in the field of regenerative medicine.

## 5 Different tissue sources of MSCs and their characteristics

### 5.1 Bone marrow mesenchymal stem cells (BM-MSCs)

Bone marrow-derived MSCs were the first MSCs to be described, known as BM-MSCs. BM-MSCs can be isolated from the sternum, vertebrae, iliac crest, and femoral shaft (123–125). Compared to other types of MSCs, the process of obtaining BM-MSCs is invasive. BM-MSCs are collected through specific bone marrow aspiration, and stem cell isolation can be quickly and efficiently performed using flow cytometry and MACS technology based on the expression of cell surface markers (126, 127). In addition to meeting the ISCT standards, BM-MSCs express other surface markers, such as STRO-1, CD146, SSEA-4, CD271, MSCA-1, and PDGFRα (128, 129). Among these, STRO-1 is highly expressed in fresh and young BM-MSCs and decreases during expansion cultures, making STRO-1-positive BM-MSCs suitable for clinical applications (129). BM-MSCs exist in very low quantities in bone marrow tissue and undergo rapid proliferation after seven days of culture, achieving a 500-fold expansion in 50 passages (130). Under phase-contrast microscopy, the dominant cells appear spindle-shaped or pool-like with abundant cytoplasm and large, dark nucleoli. After *in vitro* expansion, a sufficient number of cells can be obtained for therapeutic doses.

### 5.2 Adipose-derived mesenchymal stem cells (A-MSCs)

A-MSCs were first isolated from human adipose tissue through plastic adherence before MSCs were formally defined (131). In 2001, Zuk et al. identified MSCs in human adipose tissue and characterized them (132), which led to the recognition that MSCs isolated from adipose tissue could serve as an alternative to BM-MSCs. Adipose tissue consists mainly of adipocytes organized into lobules. It is a highly complex tissue composed of mature adipocytes that account for over 90% of the tissue volume, along with a heterogeneous group of cells surrounding and supporting the adipocytes. After enzymatic digestion and/or mechanical disruption, single-cell populations can be released from adipose tissue, forming the stromal vascular fraction (SVF) (133). Centrifugation separates the SVF from mature adipocytes. The supernatant contains low-density, floating adipocytes, while the SVF forms a denser cell pellet. The SVF is a heterogeneous mixture of various cell populations with different levels of maturity and function, including pericytes, smooth muscle cells, and A-MSCs (134). A-MSCs are further isolated from the SVF through expansion and plastic adherence, similar to BM-MSCs, to deplete most hematopoietic cell populations. Factors such as donor age, donor BMI, tissue type (white or brown adipose tissue), and tissue harvesting procedures and locations can affect the yield, proliferation rate, and differentiation capacity of A-MSCs (134–138). Compared to A-MSCs from older patients, A-MSCs from younger patients exhibit higher proliferation rates and more effectively differentiate into mature adipocytes (134). Increased BMI reduces A-MSCs proliferation and may impair *in vitro* osteogenic potential (135). Although there are two types of adipose tissue (brown and white), white adipose tissue is the source of A-MSCs used in most studies (136). Additionally, ultrasound-assisted liposuction reduces the proliferation frequency of A-MSCs and prolongs their population doubling time compared to excision methods (137). The yield of A-MSCs also depends on the tissue harvest site, with the abdomen being more suitable for A-MSCs collection than the hip/thigh region (138). A-MSCs possess the same characteristics as standard MSCs, meeting ISCT criteria. However, CD34 expression has been a unique feature of A-MSCs, with studies showing that a significant portion of A-MSCs are CD34-positive, although negative results for CD34 have also been reported (139). Evidence suggests that CD34 is expressed in tissue-resident A-MSCs, with negative findings being the result of cell culture, indicating that CD34 expression may be reversible (140). A report by Suga et al. indicated that CD34 expression is associated with immature, pro-angiogenic gene expression and greater replicative potential (141). It is speculated that CD34 is a niche-specific marker of immature/early progenitor cells that is lost under *in vitro* conditions (133, 142).

### 5.3 Placenta-derived mesenchymal stem cells (P-MSCs)

The placenta is a transient maternal-fetal organ that is highly abundant and easily accessible. Since it is discarded after delivery, its collection does not involve invasive procedures, making it more ethically acceptable. This has garnered significant interest in the placenta as a source of MSCs. P-MSCs can be isolated from various parts of the placenta, such as the chorion, basal decidua, top decidua, amnion, and amniotic fluid (143–146). P-MSCs are typically isolated using explant or enzymatic methods. In the explant method, tissue is dissected from the respective source, washed with cold phosphate-buffered saline to remove excess blood, minced into small pieces, and allowed to adhere to culture dishes. The plates are incubated at 37 °C with MSCs proliferation medium. In the enzymatic method, the tissue is similarly minced and treated with trypsin, DNase, and collagenase. The isolated cells are then plated and maintained similarly to explant cultures. P-MSCs exhibit high immunoregulatory properties by secreting soluble factors and modulating the immune functions of macrophages and dendritic cells, thereby inhibiting T cell activity and enhancing their immunosuppressive capabilities (144). Current literature suggests that P-MSCs hold great potential as a cell source for therapeutic angiogenesis. P-MSCs secrete a large number of cytokines and chemokines crucial for angiogenic signaling, promoting angiogenesis by modulating niche cells (147).

### 5.4 Umbilical cord mesenchymal stem cells (HUC-MSCs)

The human umbilical cord is an extra-embryonic connective tissue located between the mother and fetus, consisting of two arteries, one vein, vascular connective tissue, and umbilical epithelium. The connective tissue, also known as Wharton's jelly, is a spongy structure woven from collagen fibers, proteoglycans, and embedded stromal cells. The umbilical cord is a rich source of MSCs, with minimal ethical concerns. Its collection and isolation process is painless, minimally invasive, and the tissue is easy to store and transport (148). HUC-MSCs can be isolated from different parts of the umbilical cord, including blood, endothelial cells of the umbilical vein, and Wharton's jelly (149). Similar to P-MSCs, HUC-MSCs are typically isolated using explant or enzymatic methods (150). The surface markers of HUC-MSCs are similar to those defined by the ISCT for MSCs, although they are negative for CD133 (151). The phenotypic characteristics of HUC-MSCs may be influenced by the number of culture passages, culture medium, and methods used. HUC-MSCs can exert immunosuppressive effects by inhibiting the proliferation of T cells, B cells, and NK cells, and by directing monocytes and dendritic cells towards an immature state (152, 153).

### 5.5 Comparison of MSCs from four sources

Flow cytometric analysis using MSCs from different tissues revealed that all cell types exhibit similar immunophenotypes. MSCs are positive for CD29, CD44, CD73, CD90, and CD105, which are known MSC markers, with positive markers expressed across all cell types (154). These results confirm that MSCs from different sources express the surface markers defined by the ISCT. However, CD90, a typical MSC marker, is less pronounced in P-MSCs compared to other cells (154). In addition to surface markers, MSCs from different tissue sources have varying lineage differentiation capabilities. When MSCs from bone marrow, umbilical cord, adipose tissue, and placenta are cultured *in vitro*, BM-MSCs exhibit a longer population doubling time than other MSC types (154). P-MSCs can undergo more passages compared to other MSC types during passaging (154). In colony-forming unit (CFU) assays, BM-MSCs and HUC-MSCs produce more colonies than other MSC types when an equal number of MSCs are plated, indicating stronger self-renewal characteristics (154). After differentiation, BM-MSCs and AT-MSCs exhibit significant differentiation potential, generating osteoblasts, adipocytes, and chondrocytes, whereas MSCs derived from umbilical cord blood and placenta show weaker differentiation potential (154).

## 6 Mesenchymal stem cells (MSCs) and cardiomyocytes

The clinical application of MSCs began in the 1990s, initially focusing on the treatment of tumors, bone, and cartilage diseases, and demonstrated promising results. Studies have also shown the therapeutic potential and safety of using MSCs to treat various diseases. MSCs have gradually attracted attention from researchers in the field of cardiology. In preclinical models, MSCs have been used to improve heart injuries, and recent clinical trials on the safety and efficacy of MSCs therapies for heart injuries have yielded exciting and positive results. The mechanisms through which MSCs therapies benefit the heart are becoming clearer. Extensive research has shown that the beneficial effects of MSCs are not solely due to their ability to promote cardiomyocyte regeneration, which contributes only minimally. More importantly, MSCs exert paracrine effects, mediating immunomodulation and anti-inflammatory responses, thereby protecting the heart and promoting repair, while also influencing ECM remodeling favorably.

### 6.1 MSCs promote cardiomyocyte differentiation and regeneration

Early studies on the differentiation of MSCs into cardiomyocytes demonstrated that specific microenvironmental conditions, such as treatment with 5-azacytidine and platelet-derived growth factor-β, could induce MSCs differentiation into cardiomyocytes (155, 156). However, in another study, rat BM-MSCs failed to differentiate into cardiomyocytes following 5-azacytidine treatment (157). In fact, the regulatory mechanisms of MSCs multipotency are highly complex. Nevertheless, increasing evidence suggests that the activation or inhibition of certain transcription factors can guide MSCs towards specific lineage differentiation. Through exogenous overexpression or knockout of candidate genes, some key transcription factors, such as GATA4, Nkx2.5, myocardin, thioredoxin-1, and Notch1, can specifically promote the differentiation of MSCs into cardiomyocytes expressing ANP and cTnI (158, 159). Additionally, pre-treatment with TGF-β1 has been shown to enhance the differentiation of rat BM-MSCs into cardiomyocytes and improve cardiac function in a rat heart failure model (160). For stable and efficient cardiomyocyte generation from MSCs, co-treatment with 5-Aza, BMP-2, and DMSO can induce MSCs differentiation into cardiomyocyte-like cells within 24 h (161). Recent studies have shown that human amniotic MSCs (hAMSCs) can also be chemically induced to differentiate into cardiomyocyte-like cells *in vitro* using a chemical induction protocol that includes BMP4, Activin A, 5-azacytidine, CHIR99021, and IWP2 (162). These studies were conducted *in vitro*, but MSCs can also differentiate into functional cardiomyocytes *in vivo* under appropriate conditions. For instance, when human MSCs expressing the β-galactosidase reporter gene were transplanted into immunodeficient mice with experimental myocardial injury, β-galactosidase-expressing cardiomyocytes were found in the host myocardium, indicating that xenografted MSCs had differentiated into cardiomyocytes (163). Moreover, in gender-mismatched cell therapy experiments using porcine models of acute and chronic myocardial infarction, transplanted male MSCs were found to differentiate into cardiomyocytes in female host myocardium. This was confirmed by the co-localization of the Y chromosome with the cardiac transcription factors GATA-4, Nkx2.5, and the structural cardiac protein α-sarcomeric actin (164–166). These findings suggest the potential for MSCs to differentiate into cardiomyocytes *in vivo*.

#### 6.1.1 Controversies and limitations of differentiation efficiency

Although multiple studies in rodent and porcine models have confirmed the potential of MSCs to differentiate into cardiomyocytes, their differentiation efficiency remains extremely low (engraftment rate <1%, differentiation proportion 10%–14%), with marked interspecies variability and instability of long-term engraftment. For instance, using β-galactosidase labeling, Toma et al. reported that only 0.44% of human MSCs injected into mouse hearts engrafted into myocardial tissue, and merely a small proportion of these cells exhibited cardiomyocyte-like phenotypes by expressing markers such as desmin, α-actin, and cardiac troponin T (163). In a porcine model of chronic ischemic cardiomyopathy, Y chromosome tracking revealed that only 14% of the engrafted MSCs expressed cardiac-specific markers (α-sarcomeric actin, troponin T, and transcription factors GATA-4/Nkx2.5), whereas 76% of the engrafted cells remained in an immature mesenchymal state (164). Species-specific differences further constrain their differentiation potential; in canine models, MSCs differentiated exclusively into vascular-like cells, with no detectable co-localization of cardiac troponin I (167). More critically, Dixon et al. demonstrated in a sheep myocardial infarction model that although transplanted MSCs were detectable within 1 h, they completely disappeared by week eight (Y chromosome PCR negative), indicating a fundamental deficiency in their capacity for long-term engraftment and integration (168).

Collectively, these findings suggest that the contribution of MSCs to cardiac functional improvement via direct differentiation into cardiomyocytes is minimal. Recent studies increasingly support the notion that the therapeutic effects of MSCs are primarily mediated through their robust paracrine activity—by secreting a repertoire of bioactive factors, including cytokines, growth factors, and extracellular vesicles (EVs), MSCs modulate the immune microenvironment, suppress inflammation and fibrosis, promote angiogenesis, and activate endogenous repair pathways. This paradigm shift provides a novel theoretical framework for optimizing stem cell-based therapeutic strategies.

### 6.2 The paracrine role of MSCs

Although MSCs can exert beneficial effects on the heart through various mechanisms, the paracrine activity of MSCs has recently become a focal point of therapeutic research. Multiple growth factors and cytokines are secreted together as part of the “secretome.” These factors can be water-soluble or encapsulated in EVs or exosomes cultured in “conditioned media.” The secreted paracrine factors have diverse effects, such as antifibrotic, anti-apoptotic, and angiogenic properties, which improve local vascularization and reduce cardiomyocyte death. The paracrine function of MSCs is primarily attributed to their derived exosomes, which will be discussed in detail later.

## 7 Exosomes derived from mesenchymal stem cells

### 7.1 Brief introduction to exosomes

Exosomes are EVs with diameters ranging from 40 to 160 nm (169). They are secreted by various cell types, including dendritic cells, mast cells, platelets, and MSCs, and are present in most human and animal body fluids, such as plasma, serum, saliva, amniotic fluid, breast milk, and urine (170). Exosomes were first discovered by Pan and Johnstone while studying how sheep reticulocytes mature into red blood cells (171). They carry crucial information and macromolecules from their source cells, playing a vital role in intercellular communication. These macromolecules, which include various proteins, enzymes, transcription factors, lipids, ECM proteins, receptors, and nucleic acids, can be found inside or on the surface of exosomes (170). Initially, exosomes were considered to be cellular debris or waste and were thought to be indicators of cell death (172, 173). Since their discovery, the biological properties, functions, and potential clinical applications of exosomes have been extensively studied. Typical biomarkers of exosomes include proteins such as CD9, CD81, CD63, ceramide, tumor susceptibility gene 101 (TSG101), and apoptosis-linked gene 2-interacting protein X (ALIX), all of which are involved in exosome origin and biogenesis (170, 174). The most common and traditional method for isolating exosomes is ultracentrifugation/differential ultracentrifugation, which separates exosomes based on size and density (175). Other methods include ultrafiltration, size-exclusion chromatography (SEC), and molecular weight-based exosome separation (175). Immunoaffinity chromatography and precipitation can also be used to enhance the purity of isolated exosomes (174).

**Figure 1:**
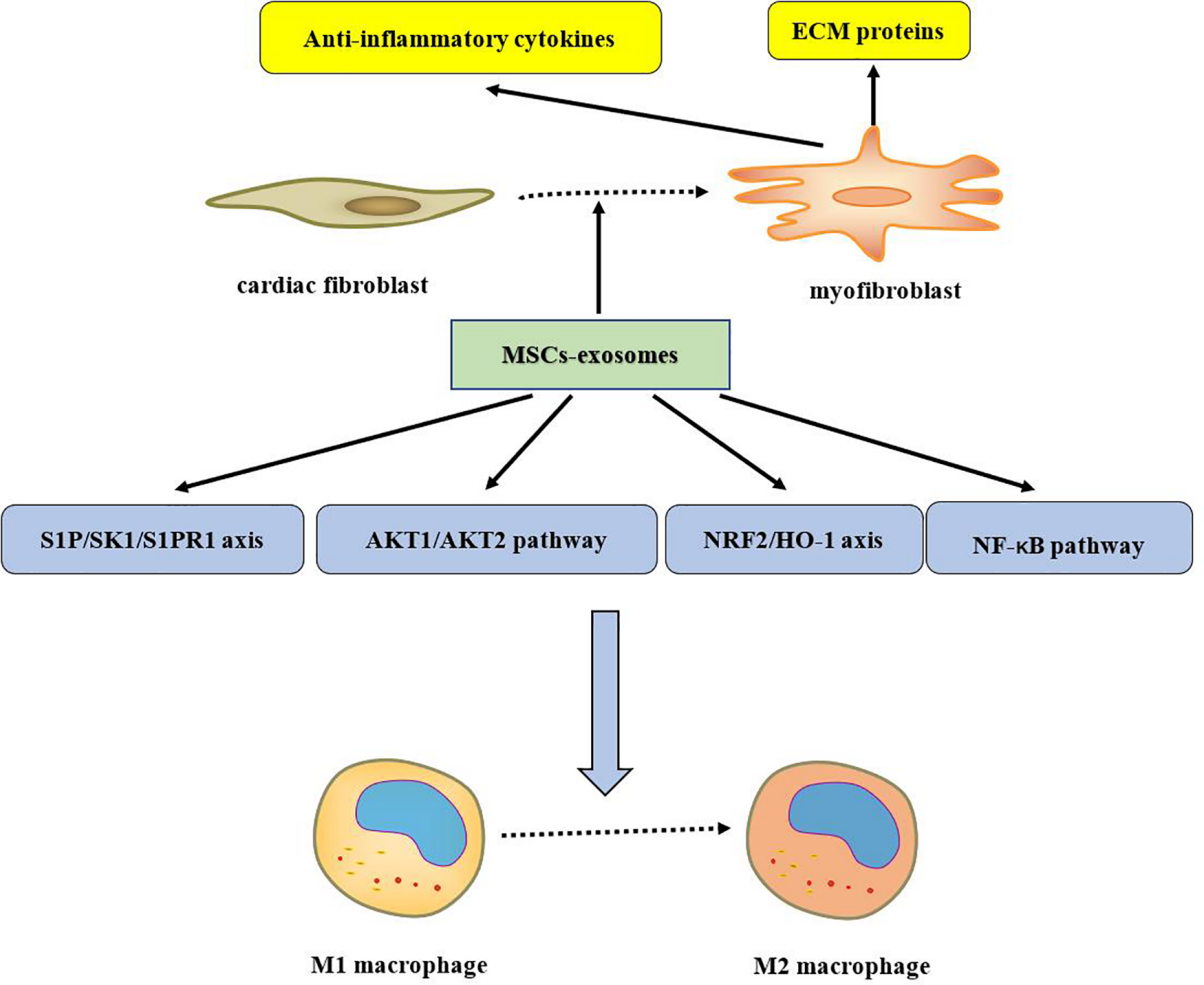
ECM, extracellular matrix; MSCs, mesenchymal stem cells; S1P/SK1/S1PR1, sphingosine-1-phosphate/sphingosine kinase 1/sphingosine-1-phosphate receptor 1; AKT, Ak strain transforming; NRF2/HO-1, nuclear factor erythroid 2-related factor 2/heme oxygenase-1; NF-*κ*B, nuclear factor kappa-B.

**Figure 2:**
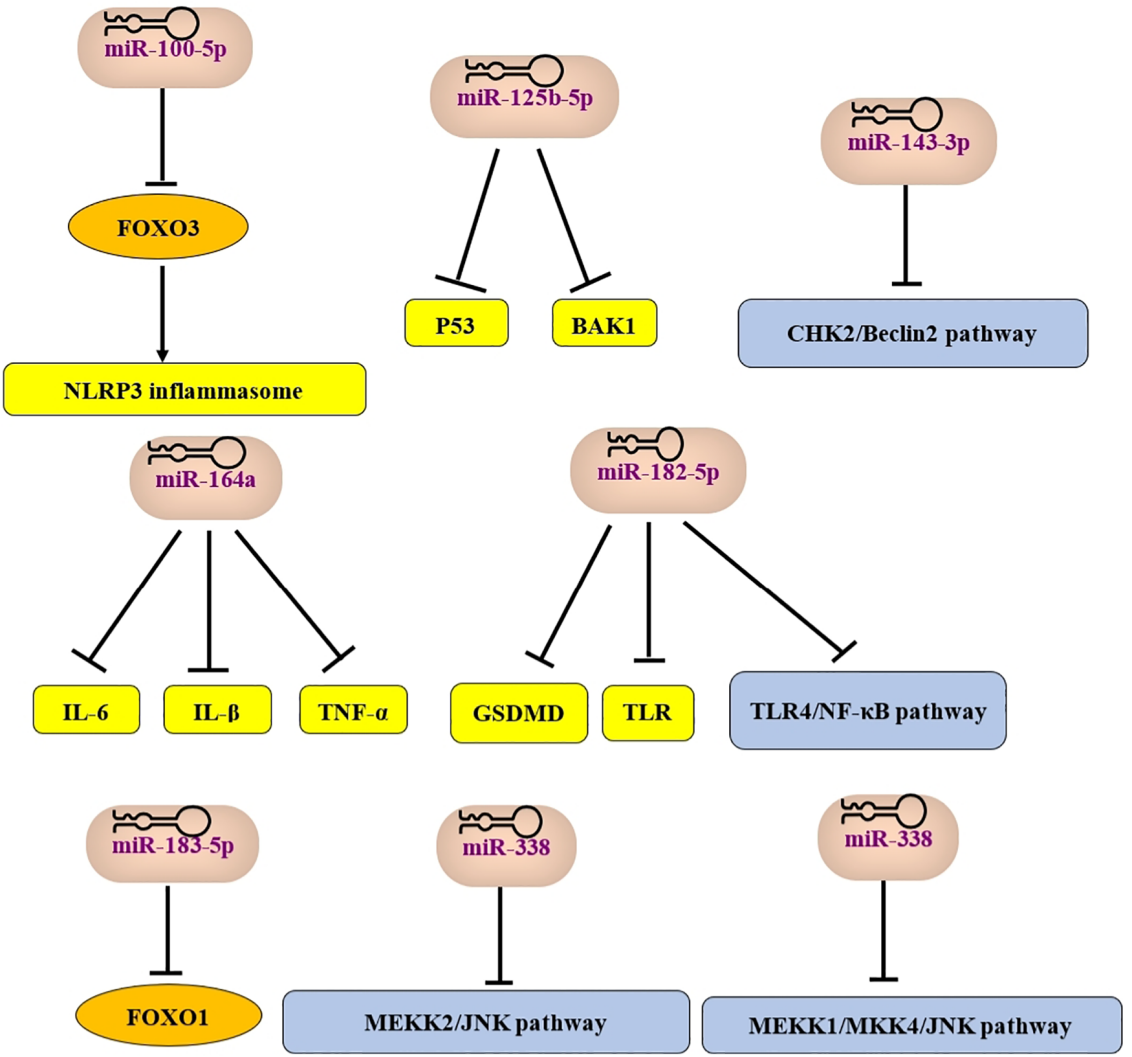
Mir, microRNA; FOXO, Forkhead box O; BAK, Bcl-2 homologous antagonist/killer; CHK2, checkpoint Kinase 2; IL, interleukin; TNF-α, tumor necrosis factor-α; GSDMD, gasdermin D; TLR, toll-like receptor; MEKK, Mitogen-Activated Protein Kinase Kinase Kinase 2; JNK, c-Jun N-terminal Kinase.

**Figure 3:**
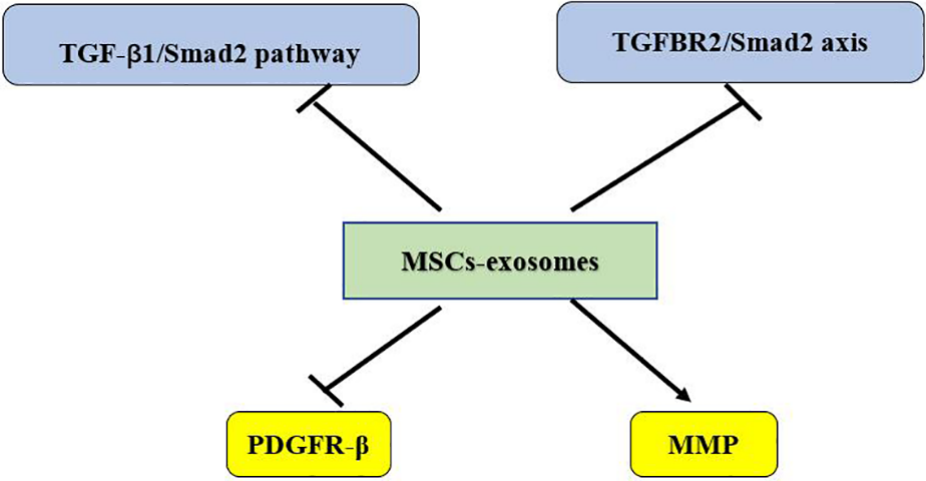
TGF, transforming growth factor; TGFBR2, transforming growth factor Beta receptor type II; PDGFR, platelet-derived growth factor receptor; MMP, matrix metalloproteinase.

**Figure 4:**
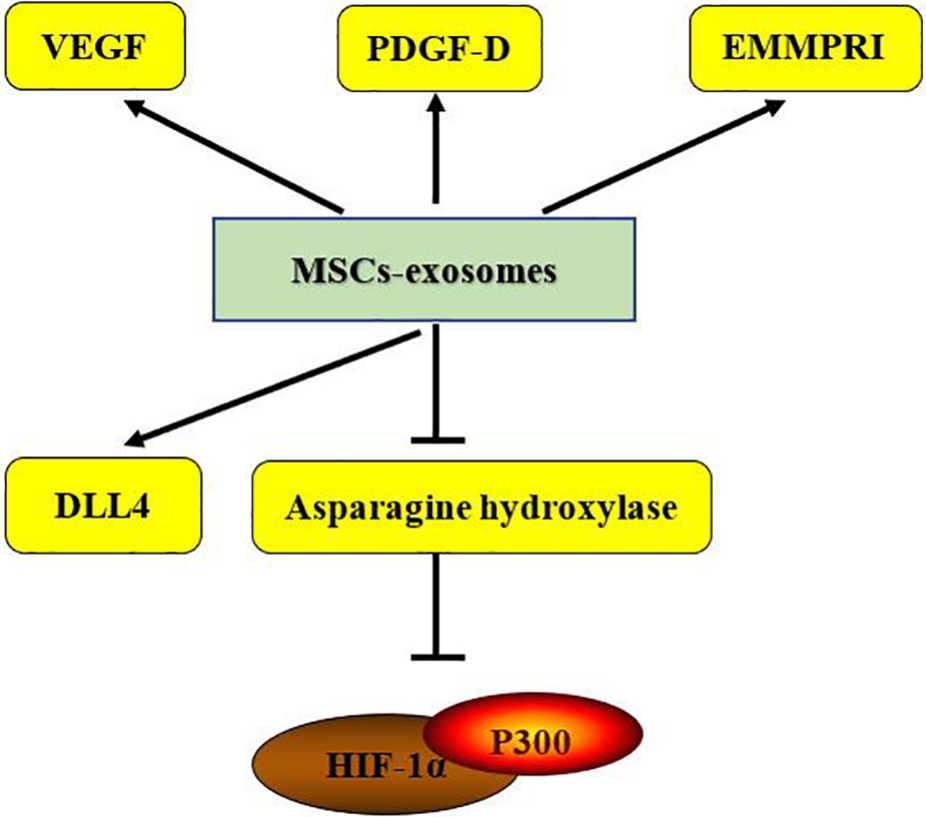
VEGF, vascular endothelial growth factor; PDGF, platelet-derived growth factor; EMMPRIN, extracellular matrix metalloproteinase inducer; DLL-4, delta-like 4; HIF, hypoxia-inducible factor.

MSCs promote angiogenesis and possess the ability to restore ischemic tissues, making them highly attractive for treating cardiovascular diseases (176). The beneficial effects of MSCs in tissue repair are largely attributed to paracrine signaling, including secreted vesicles, mainly exosomes (177). Since exosomes contain various regulatory RNAs, they have the potential to reduce tissue damage after myocardial infarction (178). Exosomes derived from different MSCs sources generally have common therapeutic effects, though their specific mechanisms vary slightly, likely due to differences in their microRNA (miRNA) content (179, 180). miRNAs, important gene expression regulators, are abundant in exosomes. They can suppress the expression of pro-apoptotic proteins, increase anti-apoptotic protein expression, improve angiogenic gene expression, mitigate oxidative stress, limit the proliferation of cardiac fibroblasts, create an anti-inflammatory microenvironment, and enhance cardiac function. These effects promote cardiomyocyte survival and improved function in various myocardial infarction or heart failure models (178, 181). Interestingly, recent studies have demonstrated that exosomes secreted by MSCs can replace MSCs-based stem cell therapy in various injury and disease models (182, 183). For example, MSC-derived exosomes (MSC-Exos) have been shown to induce repair in mouse models of wound healing and myocardial infarction (184–186). Our next aim is to elucidate the molecular mechanisms of MSC-derived exosomes to facilitate their clinical translation (as shown in Figures 1–4).

### 7.2 Exosomes and cardiomyocytes

#### 7.2.1 Cardioprotective effects

(The cardioprotective effects of MSCs-exosome are shown in Figure 1, where miRNA effects are shown in Figure 2).

Cardiomyocytes have a high oxygen demand, making them highly susceptible to hypoxia. During hypoxia and ischemia, cardiomyocytes are prone to apoptosis, oxidative stress, and excessive autophagy activation, which ultimately leads to impaired cardiac function. Increasing research has shown that exosomes derived from MSCs play a crucial role in anti-apoptotic, anti-oxidative stress, and autophagy regulation mechanisms.

Myocardial ischemia/reperfusion (I/R) injury can exacerbate apoptosis, necrosis, and cell death (187). During myocardial I/R injury, cardiomyocyte death activates inflammatory responses, which significantly impact the extent of myocardial damage. The activation of inflammatory cytokines can induce pyroptosis, leading to the release of intracellular contents into the extracellular environment. This, in turn, triggers further inflammatory responses, exacerbating myocardial damage in a vicious cycle (188). Prolonged or excessive inflammation degrades the ECM, leading to ventricular remodeling and eventually heart failure. Exosomes have been shown to inhibit inflammation by downregulating inflammatory cytokine expression and promoting the transition of macrophages from the pro-inflammatory M1 phenotype to the anti-inflammatory M2 phenotype. Macrophages play a critical role in both the progression and resolution of inflammation. M1 macrophages secrete large amounts of pro-inflammatory factors, while M2 macrophages produce various anti-inflammatory factors and growth factors (189). After myocardial injury, M1 macrophages are recruited to the damaged myocardium, exhibiting strong phagocytic and pro-inflammatory activities, after which M2 macrophages dominate to resolve inflammation and promote myocardial repair (190). Studies have shown that exosomes derived from BM-MSCs activate the AKT1/AKT2 signaling pathway and the nuclear factor erythroid 2-related factor 2/heme oxygenase-1 (NRF2/HO-1) axis, while inhibiting the NF-*κ*B signaling pathway. This signaling shift promotes macrophage polarization from the M1 to the M2 phenotype, aiding in the resolution of post-myocardial injury inflammation (191, 192). Treating BM-MSCs with fibronectin type III domain-containing protein 5 (FNDC5) can further enhance the ability of exosomes to promote macrophage polarization (192). Similar to BM-MSC-derived exosomes, exosomes from A-MSCs also facilitate the M1-to-M2 transition and suppress inflammation after myocardial injury. However, the mechanism may differ, as this effect is achieved through the activation of the sphingosine-1-phosphate/sphingosine kinase 1/sphingosine-1-phosphate receptor 1 (S1P/SK1/S1PR1) axis (193). Not all exosomes regulate inflammation through macrophage polarization. For example, HUC-MSCs exosomes exert anti-inflammatory effects via cardiac fibroblasts. After myocardial injury, HUC-MSC-derived exosomes promote the differentiation of CFs into myofibroblasts, which secrete anti-inflammatory cytokines and ECM proteins, thereby reducing inflammation and stabilizing cardiac tissue (194).

MiRNAs in exosomes play a critical role in cardioprotection. MiRNAs within exosomes derived from BM-MSCs can coordinate the suppression of genes that drive cardiomyocyte apoptosis and cardiac injury. For example, in a mouse model of myocardial infarction, hypoxia-derived exosomal miR-125b-5p exhibited cardioprotective functions by inhibiting the expression of apoptotic genes p53 and BAK1, thereby enhancing cardiac repair and reducing cardiomyocyte apoptosis (195). Exosomes from BM-MSCs carrying miR-183-5p can inhibit the expression of FOXO1, thus reducing cardiomyocyte apoptosis and oxidative stress (196). Exosomal miR-182-5p from BM-MSCs suppresses the expression of the pyroptosis-related protein gasdermin D (GSDMD), toll-like receptor 4 (TLR4), and the TLR4/NF-*κ*B signaling pathway, thereby exhibiting strong anti-inflammatory and anti-pyroptotic effects (197, 198). Notably, BM-MSC-derived exosomes contain miRNAs capable of regulating signaling pathways. For instance, miR-338 alleviates cardiomyocyte apoptosis via the MEKK2/JNK pathway (199), while miR-455-3p inhibits the MEKK1-MKK4-JNK signaling pathway (200). Furthermore, miR-143-3p regulates autophagy by inhibiting the CHK2/Beclin2 pathway, effectively suppressing cardiomyocyte apoptosis (201). Exosomes derived from miR-146a-modified A-MSCs have been shown to reduce local inflammation by inhibiting the release of pro-inflammatory factors (IL-6, IL-1β, and TNF-α), thus alleviating myocardial injury (202). The same study reported that exosomes from A-MSCs improve cardiac function by preventing apoptosis through downregulation of early growth response factor 1 (EGR1) (202). Research has also demonstrated that HUC-MSC-derived exosomes provide additional cardioprotection by promoting the expression of Smad7 through the inhibition of miR-125b-5p in the injured myocardium (203). Additionally, HUC-MSC-derived exosomes containing miR-100-5p can downregulate FOXO3, thereby inhibiting NLRP3 inflammasome activation and protecting cardiomyocytes from pyroptosis and damage (204).

#### 7.2.2 Anti-myocardial fibrosis

(The antimyocardial fibrosis effect of MSCs-exosome is shown in Figure 3).

Following myocardial injury, ischemia and cell death of cardiomyocytes trigger various repair responses, among which myocardial fibrosis is a critical process. Although the initial fibrotic response helps prevent ventricular wall rupture after myocardial infarction, excessive fibrosis leads to progressive cardiac dysfunction and eventually heart failure. Therefore, reducing excessive myocardial fibrosis is a key research focus for improving heart failure outcomes.

Recent studies have demonstrated that MSCs-derived exosomes can significantly reduce infarct size, fibrosis area, and cardiomyocyte apoptosis in models of MI. Compared with control-treated infarcted pigs, intravenous administration of exosomes within 7 days resulted in a marked reduction in infarct size and preservation of cardiac function (205). Moreover, in mouse models of MI, exosome-treated mice exhibited higher survival rates. MSC-derived exosome treatment significantly decreased leukocyte accumulation in and around the infarct zone, as well as the expression of LOX-1, NLRP3 inflammasomes, and cell death markers (206). The underlying mechanism involves the inhibition of fibroblast-to-myofibroblast transformation mediated by TGF-β, thus reducing myocardial fibrosis. Further research has shown that MSC-derived exosomes inhibit the TGF-β1/Smad2 signaling pathway to alleviate myocardial injury and fibrosis (207). Exosomes from A-MSCs carry miR-671, which reduces myocardial fibrosis and inflammation by inactivating the TGFBR2/Smad2 axis (208). BM-MSC-derived exosomes upregulate PDGFR-β, enhancing microvascular regeneration, inhibiting fibrosis, and maintaining long-term cardiac function after I/R injury (209). Notably, BM-MSC-derived exosomes exhibit better therapeutic efficacy than BM-MSCs themselves, indicating their potential for clinical application in future stem cell transplantation technologie (209). The remodeling of ECM plays a crucial role in the development of ventricular dilation and heart failure. Among the many proteases present in cardiac ECM, MMPs drive the degradation of cardiac matrix during remodeling. Increased MMP activity may lead to collagen degradation, heightened inflammation, ECM remodeling, and progressive ventricular dilation and dysfunction (210). Exosomes derived from U-MSCs have been reported to contain TIMP2, an MMP inhibitor, which suppresses ECM remodeling and collagen deposition, thereby preventing adverse cardiac remodeling (187).

#### 7.2.3 Promotion of angiogenesis

(The vascular regeneration-promoting effects of the MSCs-exosome are shown in Figure 4.)

After myocardial injury, the ability of the affected region to generate new blood vessels is limited. Severe impairments in angiogenesis can exacerbate myocardial hypoxia, further aggravating myocardial injury. Insufficient capillary formation during heart failure promotes the transition from compensatory hypertrophy to decompensation, eventually leading to heart failure (211). This process is closely linked to the regulation of angiogenesis by endothelial cells. Cardiac endothelial cells, when stimulated by angiogenic factors such as vascular endothelial growth factor (VEGF), platelet-derived growth factor, and fibroblast growth factor, activate angiogenic signaling pathways, promoting angiogenesis (212). When the secretion of these angiogenic factors is insufficient, endothelial cell proliferation and migration are suppressed, worsening the lack of angiogenesis and heart failure (213). Studies have shown that exosomes derived from human amniotic fluid mesenchymal stem cells (hAFMSC-Exos) promote angiogenesis in endothelial cells and enhance vascular formation during cardiac fibrosis, potentially through the upregulation of VEGF expression (214). Other studies indicate that exosomes from HUC-MSCs improve angiogenesis and cardiac function by activating platelet-derived growth factor D (PDGF-D) (215). These findings suggest that MSC-derived exosomes can stimulate endothelial cells by promoting the secretion of angiogenic factors, thereby enhancing angiogenesis. In addition, exosomes secreted by adipose-derived MSCs (A-MSC-Exo) transfer miR-125a to endothelial cells, promoting angiogenesis by inhibiting the angiogenesis inhibitor delta-like 4 (DLL4) (216). Thus, A-MSC-Exo may serve as a novel angiogenic factor and a potential candidate for heart failure therapy. Previous studies have shown that hypoxia-inducible factor 1-α (HIF-1α) overexpressing exosomes derived from mesenchymal stem cells rescued the impaired angiogenic capacity, migration, and proliferation of hypoxia-pretreated human umbilical vein endothelial cells *in vitro* and mediated cardioprotection through the up-regulation of pro-angiogenic factors and enhancement of neointima formation (217). Subsequently, exosomes extracted from A-MSCs were found to contain miR-31, which down-regulates asparagine hydroxylase, an enzyme that blocks the binding of HIF-1α to the coactivator p300, thereby “rescuing” angiogenic gene expression (218). In addition, ECM metalloproteinase inducer (EMMPRIN) has been shown to promote angiogenesis through hypoxia-inducible factor-2α (HIF-2α)-mediated regulation of soluble vascular endothelial growth factor isoforms and its receptor VEGFR-2 (219). Another study showed that MSC-derived exosomes contain comparable levels of VEGF and EMMPRIN, and MSC-derived exosomes can stimulate angiogenesis via EMMPRIN (220).

## 8 Existing challenges and limitations of MSCs and their exosomes in heart failure therapy

MSCs and their exosomes have emerged as promising therapeutic approaches for HF, with several clinical trials demonstrating encouraging results. However, significant challenges remain. These limitations can be broadly categorized into four areas: (1) heterogeneity in therapeutic efficacy and unclear mechanisms of action, (2) lack of standardized protocols for clinical translation, (3) concerns regarding long-term safety and production quality, and (4) obstacles in implementing individualized therapy.

### 8.1 Heterogeneity in efficacy and unclear mechanisms

One of the major challenges in MSCs-based therapy for HF is the pronounced heterogeneity in treatment outcomes and the incomplete understanding of the underlying mechanisms. Numerous clinical trials have reported variable results, likely stemming from a complex interplay among factors such as cell processing methods, therapeutic regimens, and patient-specific pathological conditions. For instance, the C-CURE trial utilized bone marrow-derived MSCs preconditioned with a cardiogenic cocktail, leading to notable improvements including a 7% increase in LVEF, a 24.8 ml reduction in end-systolic volume, and a 62-m gain in 6-min walk distance (221). In contrast, the CHART-1 trial, which employed a fixed high-dose regimen of bone marrow-derived MSCs in end-stage HF patients, failed to meet its primary endpoints and showed only limited benefit in certain subgroups (222, 223). Further analysis suggests that the cardiopoietic preconditioning strategy in C-CURE may have significantly enhanced the reparative potential of the cells, while individualized dosing based on body weight may be more advantageous than fixed dosing schemes. Additionally, the pathological microenvironment in end-stage HF may compromise the viability and functionality of transplanted cells.

Significant variation in therapeutic efficacy is also observed among MSCs derived from different tissue sources. In the RIMECARD trial, umbilical cord-derived MSCs (UC-MSCs) led to greater improvements in LVEF compared with placebo, and UC-MSCs were found to express hepatocyte growth factor (HGF) at levels 55 times higher than bone marrow-derived MSCs, suggesting that differences in the paracrine profiles of MSCs may influence therapeutic outcomes (224). The MSC-HF trial highlighted inter-individual variability in the effectiveness of autologous bone marrow-derived MSCs, which may be attributable to baseline patient characteristics such as age, comorbidities, and the etiology of heart failure (225). Notably, the POSEIDON trial found spatially specific effects of MSCs in improving regional contractile function within scarred myocardial zones, indicating that the local myocardial microenvironment may critically influence therapeutic efficacy (226).

Although the prevailing view is that MSCs exert their therapeutic benefits mainly through paracrine mechanisms, the precise molecular pathways involved remain poorly understood. The CONCERT-HF trial revealed cell-type-specific therapeutic effects: MSCs primarily improved patient-reported quality of life, whereas c-kit-positive cardiac progenitor cells (CPCs) demonstrated superiority in reducing major adverse cardiovascular events—a difference whose mechanism remains to be elucidated (227). While HGF is considered a key effector molecule, the downstream signaling pathways by which it orchestrates antifibrotic and proangiogenic responses require further clarification. Exosomes, as crucial paracrine mediators, pose additional challenges due to their compositional complexity, and current methodologies lack the sensitivity and specificity to reliably identify the key bioactive components responsible for therapeutic effects. These mechanistic uncertainties hamper the optimization and broader clinical application of MSCs-based therapies.

### 8.2 Challenges in standardization for clinical translation

The clinical translation of MSCs therapy for HF faces numerous challenges, among which the standardization of cell preparation processes is particularly prominent. Significant variability exists in the cell culture systems used across clinical trials, directly impacting cell quality and therapeutic outcomes. For instance, in the MSC-HF trial, the average culture period was 46.9 ± 10.5 days (225), whereas the C-CURE trial required an additional 4–6 weeks of cardiopoietic preconditioning. Furthermore, only 75% of patients in C-CURE met the release criteria (>600 × 10^6^ cells) (221). In addition, there is no standardized method for exosome isolation; ultracentrifugation and commercial kits vary in yield, purity, and bioactivity. Optimal dosage and delivery routes (e.g., local injection vs. systemic administration) also remain controversial (228, 229). In the CONCERT-HF trial, 25% of MSCs and 16% of CPCs were excluded due to insufficient cell quantity or activity (227), highlighting the sensitivity of the manufacturing process and the critical importance of quality control.

In terms of delivery techniques, the demand for precision remains inadequately addressed. Intramyocardial injection methods, such as NOGA-guided endocardial delivery, are technically complex and operator-dependent. Approximately 20% of patients in the POSEIDON and CONCERT-HF trials were unable to receive the full injection protocol due to anatomical limitations (226, 227). Although the C-Cathez™ catheter used in the CHART-1 trial improved procedural efficiency, it still resulted in four cases of cardiac tamponade (222). Emerging technologies, such as real-time MRI-guided injections and robotic-assisted delivery systems, have shown promise in enhancing targeting accuracy; however, the associated costs are five to eight times higher than conventional approaches, limiting widespread adoption (230). Additionally, the application of exosomes in tissue repair is constrained by their low stability and short retention time. Studies have shown that exosomes are undetectable as early as 3 h post-myocardial injection (231). Moreover, systemic delivery faces targeting inefficiencies, with less than 1% of intravenously administered exosomes reaching the cardiac tissue (232, 233). Thus, future research should explore myocardial-specific modification strategies to improve delivery efficiency.

Another major barrier is the lack of consensus on endpoint measures, which limits comparability across clinical trials. Current studies use highly heterogeneous primary outcomes, including LVEF, left ventricular end-systolic volume (LVESV), scar size (assessed via MRI), and quality of life scores. However, some functional endpoints, such as the 6-min walk test, are susceptible to placebo effects—improvements of up to 30% have been observed in placebo groups in certain trials. Therefore, there is an urgent need to establish more objective and comprehensive composite endpoints that integrate imaging parameters (e.g., global longitudinal strain, GLS), biomarkers (e.g., NT-proBNP), and hard clinical outcomes (e.g., HF rehospitalization, cardiovascular mortality) to enhance the comparability and translational value of research findings.

### 8.3 Long-term safety and manufacturing challenges

The clinical translation of MSC therapy for HF is further hampered by long-term safety concerns and manufacturing limitations. Most current trials have relatively short follow-up durations (median 12–39 months), and potential risks such as tumorigenicity and immune rejection have yet to be fully elucidated. For example, in the RIMECARD trial, one patient treated with umbilical cord-derived MSCs was diagnosed with acute lymphoblastic leukemia 13 months later. Although trace-back analysis found no direct link to the treatment, the genomic instability that may result from replicative senescence during prolonged *in vitro* expansion of allogeneic MSCs remains a concern (224, 234, 235). While exosomes circumvent the risk of uncontrolled cell proliferation, their long-term immunomodulatory effects remain debatable. For instance, overexpression of programmed death-ligand 1 (PD-L1) may impair immune surveillance, a potential risk that warrants further validation through extended follow-up and larger sample sizes (236).

The scalability of the manufacturing process presents another critical bottleneck. Autologous MSCs therapies typically require 6–8 weeks from cell harvest to administration (as in the C-CURE trial), resulting in high costs and delayed treatment (223, 237). Donor-dependent variability in cell quality further complicates production. Compared with younger donors, MSCs from elderly patients show a 40%–60% reduction in proliferative capacity, leading to marked differences in therapeutic efficacy between batches (237–239). Although allogeneic umbilical cord-derived MSCs offer the advantage of cryopreserved “off-the-shelf” availability, post-thaw viability may decrease by 15%–30%, and the long-term effects of cryostorage on functional potency remain unclear (240). Exosome production faces its own set of technical limitations—fewer than 1 μg of exosomal protein can be harvested per milliliter of culture medium. Moreover, prolonged storage at −80 °C can compromise biological activity, hindering large-scale application (241–243).

The limitations of current quality control systems further exacerbate the complexity of clinical translation. Conventional assays (e.g., flow cytometry for phenotypic analysis) are time-consuming and insufficient for real-time clinical quality control. Although emerging technologies such as Raman spectroscopy offer real-time monitoring potential, they have not yet been standardized for clinical use (244–246). Exosome quality control is particularly challenging. Only a limited number of laboratories worldwide can simultaneously assess nanoparticle size distribution (via nanoparticle tracking analysis), purity markers (e.g., CD81/CD63), and functional potency (e.g., cell migration assays) (247, 248). Additionally, the absence of standardized functional evaluation systems—such as quantification of paracrine factors or assessments of tissue repair capacity—makes it difficult to ensure consistency between production batches (248, 249). These technical gaps not only compromise the predictability of therapeutic outcomes but also complicate regulatory approval processes. Moving forward, the development of automated quality control platforms and functional performance-based evaluation standards is crucial to overcoming current technological barriers and advancing stem cell therapies toward standardized and precise clinical application.

### 8.4 Barriers to personalized implementation

The personalized application of MSCs for heart failure remains hindered by multiple technical bottlenecks and translational challenges. Currently, patient stratification relies primarily on coarse indicators such as LVEF and the NYHA functional classification. However, *post hoc* analyses of the CHART-1 trial suggest that left ventricular end-diastolic volume (LVEDV) may serve as a more precise stratification marker (222).

The integration of multi-omics technologies offers promising avenues for precise stratification. Transcriptomic analysis has identified TNF-α as a key regulator of MSCs functionality; while TNF-α can activate the reparative and immunomodulatory capacities of MSCs via the inflammatory microenvironment, prolonged exposure or excessive concentrations may lead to functional exhaustion. Therefore, fine-tuning of TNF-α signaling is critical for optimizing MSC-based therapies (250). Proteomic profiling has revealed galectin-3 (Galectin-3) as a central mediator of the immunosuppressive and anti-inflammatory properties of MSCs, acting through regulation of macrophage polarization, T cell functionality, and fibrotic pathways (251–253). Future efforts are needed to delineate the dose-dependent effects and signaling networks of Galectin-3 to facilitate its targeted application in MSC therapy. Moreover, metabolomic data indicate that enhanced glycolysis and mitochondrial remodeling are tightly associated with the immunoregulatory function of MSCs. Targeting metabolic pathways may thus provide a novel strategy to modulate the immunosuppressive potential of MSCs (254). However, the clinical application of multi-omics technologies is limited by high per-test costs and the need for interdisciplinary expertise in data interpretation.

Dose optimization represents another critical challenge. Current preclinical studies demonstrate a non-linear, inverted U-shaped dose-response relationship, whereby both low and high doses result in inferior outcomes (255). The MSC-HF trial indicated that high doses (>100 × 10^6^ cells) significantly improved cardiac function (225), whereas the POSEIDON trial found that the 20 × 10^6^ cell group exhibited greater reduction in myocardial scar size compared to the 100 × 10^6^ cell group (226). Such discrepancies may reflect heterogeneity in the pathological microenvironment—advanced heart failure patients often experience severe myocardial hypoxia and elevated inflammatory cytokines, which may lead to increased cell apoptosis at higher doses due to intensified competition for nutrients, whereas lower doses may survive better and exert paracrine effects in less hostile environments.

The determination of optimal treatment windows poses an additional clinical challenge. Preclinical studies have demonstrated that the therapeutic window for preventing I/R injury is narrow and requires early intervention before full activation of apoptotic pathways (255). The timing of MSC administration significantly affects therapeutic efficacy, though results remain inconsistent. In large animal models, administration 30 min post-reperfusion yielded variable outcomes—some studies reported improvements in infarct size or ejection fraction, while others found no benefit (256–258). In contrast, intravenous delivery 15 min post-reperfusion or delayed injection at 3 days to 3 months post-infarction facilitated functional recovery or remodeling (165, 259), whereas administration at 30 days post-MI may expose MSCs to detrimental microenvironments that compromise cell viability (260). Clinical studies further underscore the importance of timing; meta-analyses suggest that administration within one week of myocardial infarction, particularly 5–7 days PCI, enhances ejection fraction and myocardial perfusion. In contrast, very early administration (within 24 h) may reduce infarct size without improving overall cardiac function (261).

## 9 Optimization strategies for MSCs and their exosomes in heart failure therapy

The complex pathophysiology of heart failure necessitates further enhancement of the efficacy, targeting specificity, and durability of MSCs and exosome-based therapies. Recent advances in bioengineering, nanomedicine, and preclinical research have yielded multidimensional strategies to improve therapeutic outcomes. This section systematically outlines the most promising optimization approaches, including functional enhancement, innovative delivery systems, combinatorial therapeutic strategies, standardized manufacturing, and individualized treatment pathways, while discussing their translational potential based on the latest research findings.

### 9.1 Functional enhancement: genetic regulation and engineering modification

In recent years, genetic engineering and preconditioning strategies have achieved substantial progress in enhancing the therapeutic efficacy of MSCs and their exosomes. In the domain of genetic regulation, the application of CRISPR-Cas9 and viral vector technologies has enabled precise genetic modifications of MSCs, thereby significantly improving their reparative capacity (262–265). Genetic modifications have been shown to augment MSC efficacy in heart failure by promoting cell survival, enhancing differentiation and integration, and strengthening paracrine signaling (266). For instance, overexpression of heat shock protein 27 (HSP27) and heme oxygenase-1 (HO-1) has been reported to increase MSCs survival, reduce apoptosis, and improve left ventricular function (267, 268). Furthermore, overexpression of stromal cell-derived factor 1 (SDF-1) and its receptor CXCR4 enhances MSCs homing ability, reduces ventricular remodeling, and restores cardiac function (269, 270). Similarly, upregulation of IGF-1, HGF, and Ang-1 has been shown to promote angiogenesis, attenuate infarct size, and improve cardiac performance (271–273).

Preconditioning under hypoxic conditions (0.5%–3% O_2_) has also been demonstrated to activate the PI3K-Akt signaling pathway, enhance cellular survival, and upregulate key anti-apoptotic and pro-angiogenic factors including HIF-1α, VEGF, and Bcl-2, thereby reducing infarct size and improving cardiac outcomes (274). Exosome engineering represents another promising direction. For example, exosomes modified with ischemic myocardium-targeting peptides (IMTP-exosomes) have shown superior therapeutic efficacy compared to unmodified exosomes, significantly alleviating inflammation, reducing apoptosis and fibrosis, promoting angiogenesis, and enhancing cardiac function following myocardial infarction (275). Additionally, exosome microneedle patches loaded with microRNA-29b have been found to effectively prevent post-infarction cardiac fibrosis (276). Exosomes overexpressing CD47 have demonstrated improved targeting efficiency by evading immune clearance via the “don't eat me” signal (277, 278). These strategies provide powerful tools to optimize MSCs and exosome-based therapies, although further investigations are required to address challenges in clinical translation.

### 9.2 Innovative delivery systems: biomaterials-driven precision therapeutic strategies

Although substantial progress has been made in applying MSCs and their exosomes for cardiac repair, conventional intramyocardial injection remains hindered by limited cell viability, uneven distribution, and difficulties in maintaining therapeutic function. To overcome these limitations, researchers have developed a range of biomaterial-based innovative delivery systems that improve local retention, enable controlled release, and enhance the stability of bioactive molecules, thereby significantly boosting the therapeutic potential of MSCs-based interventions.

Hydrogel scaffolds, owing to their superior biocompatibility and biomimetic properties, have emerged as one of the most promising delivery vehicles. These scaffolds mimic the native ECM microenvironment, thereby enhancing MSCs retention and functional expression (279, 280). Specifically, injectable peptide and alginate hydrogels loaded with EVs have demonstrated improved retention at the site of myocardial injury, facilitated angiogenesis, and mitigated fibrotic progression (281). The incorporation of 3D culture technologies has further potentiated the cardioprotective effects of these systems, offering new avenues for myocardial regeneration (282, 283).

Microsphere and encapsulation technologies also offer unique advantages in delivery system design. MSCs-derived microspheres formed via self-assembly into 3D structures have been shown to enhance anti-apoptotic capacity and optimize paracrine signaling (284). Spheroids cultured on chitosan membranes have exhibited enhanced myocardial contractility in animal models, potentially mediated by angiogenesis-related microRNAs (285, 286). Semipermeable encapsulation platforms provide physical protection from immune rejection while allowing the exchange of nutrients and signaling molecules, offering an ideal foundation for integrating gene editing technologies and achieving controlled release of therapeutic factors (266, 287–289).

Recently, the development of magnetically responsive delivery systems has opened new frontiers for precision therapy. MSCs labeled with superparamagnetic iron oxide nanoparticles (SPIONs) have demonstrated significantly enhanced myocardial engraftment under magnetic guidance (290). Moreover, magnetic navigation technologies have been shown to increase targeting accuracy and therapeutic efficacy of MSCs and their exosomes in heart failure models (291, 292). Future research should further investigate the interplay between biomaterials and cellular functions, aiming to develop intelligent delivery platforms with targeted homing and dynamic responsiveness. Additionally, standardization and large-scale production of these technologies will be crucial for their clinical translation, providing more effective solutions for cardiovascular therapy. These innovative strategies represent a major step toward overcoming the limitations of traditional cell therapies and highlight the vast potential of tissue engineering in regenerative medicine.

### 9.3 Combined therapies: synergistic mechanisms and clinical translation

In recent years, combination strategies involving MSCs and their exosomes have achieved significant advances in the treatment of heart failure, primarily by leveraging synergistic mechanisms to amplify therapeutic efficacy. Among these, the co-administration of cells and pharmacological agents has emerged as a promising approach. Drug preconditioning with agents such as trimetazidine, melatonin, and statins has been shown to activate the Akt signaling pathway, reduce apoptosis, promote angiogenesis, and modulate immune responses—thereby significantly enhancing MSCs survival and tissue repair capacity (293–297). Currently, a clinical trial is underway investigating the combined application of atorvastatin and autologous BM-MSCs in patients undergoing PCI, aiming to improve cell survival through pharmacological dose optimization (298).

The therapeutic synergy of combination approaches is highly dependent on temporal coordination and vector design. As discussed in the previous section, combining MSCs with biomaterials (e.g., hydrogels or cell sheet technologies) enhances cell retention and facilitates myocardial repair. Notably, in a rat model of MI, sequential delivery of MSCs and their derived exosomes has demonstrated superior therapeutic effects (299). In this study, exosomes were administered 30 min post-MI to target the acute inflammatory phase, followed by MSC transplantation on day 3 to support regenerative processes (299). This precisely timed protocol significantly reduced infarct size and improved cardiac contractility, highlighting the potential advantages of temporally coordinated cell-exosome therapy.

More complex combination strategies involve co-transplantation of MSCs with CPCs. The CONCERT-HF trial has demonstrated that while CPCs primarily reduce clinical events and MSCs improve quality of life, their combined use yields additive benefits by targeting complementary aspects of cardiac repair (227). Additionally, the co-administration of MSCs with regulatory T cells (Tregs) has been shown to enhance MSC survival, proliferation, and pro-angiogenic potential, offering a novel avenue for cell-based combinatorial therapies (300).

Despite promising preclinical results, the clinical translation of combination therapies remains challenged by the need for optimized therapeutic regimens, thorough safety evaluations, and standardized manufacturing protocols. Future efforts should focus on developing intelligent delivery systems and conducting multicenter clinical trials to facilitate the adoption of combination strategies in clinical practice.

## 10 Conclusion and future prospects

Despite significant advancements in HF treatment, the five-year mortality rate for HF remains as high as 50%, making it a major global health concern. The pursuit of more effective therapeutic strategies for HF has long been the goal of many medical professionals. The discovery of MSCs and their exosome derivatives in the field of cardiac regenerative medicine has attracted widespread attention. Current evidence suggests that the use of MSCs and their exosomes can improve the ejection fraction in HF patients, with many preclinical trials showing promising results.

Although an increasing number of clinical trials have demonstrated the significant efficacy of MSCs and their exosomes in treating HF, and they hold considerable promise for clinical applications, several challenges remain. With longer follow-up periods, it is unclear whether the benefits of stem cell therapy will persist. Furthermore, the standardization of stem cell preparation, delivery methods, and dosing regimens has yet to be established. Additionally, most current studies have small sample sizes, limited evaluation methods for cardiac function, and follow-up periods of less than two years.

Therefore, future research should involve larger patient cohorts, longer follow-up periods, and more comprehensive evaluation methods. The promotion of personalized treatment should also be applied to MSCs transplantation, tailoring the choice of delivery methods, dosing, and follow-up strategies according to individual patient differences to achieve the best therapeutic outcomes. This represents another promising direction for future research on stem cell transplantation in the treatment of HF.
